# A small, microRNA-size, ribonucleic acid regulating gene expression and development of Shiga toxin-converting bacteriophage Φ24_Β_

**DOI:** 10.1038/srep10080

**Published:** 2015-05-11

**Authors:** Bożena Nejman-Faleńczyk, Sylwia Bloch, Katarzyna Licznerska, Aleksandra Dydecka, Agnieszka Felczykowska, Gracja Topka, Alicja Węgrzyn, Grzegorz Węgrzyn

**Affiliations:** 1 Department of Molecular Biology, University of Gdansk, Wita Stwosza 59, 80-308 Gdansk, Poland; 2 Laboratory of Molecular Biology (affiliated with the University of Gdansk), Institute of Biochemistry and Biophysics, Polish Academy of Sciences, Wita Stwosza 59, 80-308 Gdansk, Poland

## Abstract

A microRNA-size (20-nt long) molecule has been identified in *Escherichia coli* after induction of Shiga toxin-converting bacteriophage Φ24_B_. This small RNA, named 24B_1, is encoded in the *lom*-*vb_24B_43* region of the phage genome, and apparently it is produced by cleavage of a larger transcript. A phage devoid of 24B_1 revealed decreased efficiency of lysogenization, quicker prophage induction after provoking the SOS response, higher efficiency of progeny phage production during the lytic cycle and less efficient adsorption on the host cells. Expression of most of phage genes was drastically increased after infection of *E. coli* by the Φ24_B_Δ24B_1 phage. Since 24B_1 may impair expression of the *d_ant* gene, coding for an anti-repressor, these results may explain the mechanism of regulations of the physiological processes by this small RNA due to impaired activity of the cI repressor and changed expression of vast majority of phage genes. To our knowledge, this is the first example of functional microRNA-size molecule in bacterial cells.

Small bacterial RNAs (sRNAs) have been extensively studied in the last decade. These regulatory molecules have been discovered to regulate different processes such as carbon metabolism, virulence, biofilm formation or response to stresses, including oxidation, iron starvation, sugar-phosphate stress or outer membrane perturbation^1^,^2^]. The bacterial small RNAs show high diversity in size and structures, and exhibit different molecular mechanisms of action. One group of bacterial sRNAs includes molecules that bind directly to proteins and affecting their activity^3^. Another group includes riboswitches which are most often located in the 5’ untranslated region (5’ UTR) of bacterial mRNA, directly interact with metabolites and control gene expression via a secondary structural switch^4^.

The best–characterized group of bacterial sRNAs acts by antisense base pairing, and can be divided into two important classes. The first class encompasses true antisense RNAs, which are synthesized from the strand complementary to the mRNA they regulate and function by base pairing with extensive complementarity with the target mRNA. The second class includes molecules that also act by pairing but have limited complementarity with their targets and usually are found at genomic locations remote from those of their targets. sRNAs from this group are the most related to eukaryotic microRNAs and siRNAs in their ability to modulate the activity and stability of multiple mRNAs^1^,^2^,^5^]. These sRNAs vary in size between 50 to 350 nucleotide in length, and unlike to eukaryotic miRNAs and siRNAs, they usually are not processed, although for a few sRNAs, cleavage does occur, and a shorter form (~20 nt) is also seen^6^,^7^].

sRNAs control gene expression posttranscriptionally via base pairing with mRNA targets leading to positive or negative regulation of target protein synthesis. In many cases, the interaction between sRNA and target mRNA require an RNA chaperone, the Hfq protein, that facilitates base pairing of the sRNA-mRNA complex and stabilizes the sRNA molecule. Positive regulation often involves sRNA base pairing to a sequence in the target mRNA that may otherwise form a translation-inhibitory secondary structure which masks the ribosome binding site (RBS) and suppress loading of ribosome. Such sRNA-mRNA pairing prevents formation of the inhibitory structure, and unmasks the RBS allowing translation of the target protein. Negative regulation may occur via different mechanisms. Interaction between sRNA and target mRNA may occlude RBS, and result in repression of translation. Sometimes the inhibition of translation is coupled to degradation of the sRNA-mRNA complex by the RNase E. In some cases, the degradation of mRNA occurs without affecting the translation process^5^,^8^,^9^]. Interestingly, sRNAs molecules which control expression of genes encoded in operons have been discovered recently. Such sRNAs may regulate all genes encoded by target polycistronic mRNA or just selected genes from the operon (for more details, refer to^5^).

Genome-wide searches in numerous microorganisms allowed to identify a large group of bacterial sRNAs. These molecules have been well studied in case of model organisms such as *Escherichia coli* and *Salmonella enterica*. The range of 80-100 sRNA molecules have been reported for *E. coli*^1^. Interestingly, microRNA-size small RNA fragments (15-28 nt) were also reported in recent studies on *E. coli*^7^, however bacterial RNAs of comparable size to eukaryotic microRNAs have received little attention up to now. In a very recent study, Furuse *et al.* found a 23-nt small RNA produced by *Mycobacterium marinum* and proposed that it can be the first discovered candidate for microRNA of bacterial origin^10^.

The sRNAs have been found in several pathogenic species of bacteria such as *Listeria* spp., *Vibrio* spp. or *Staphylococcus* spp.^11^. Moreover, their presence has been demonstrated in enterohemorrhagic *E. coli* (EHEC) strains, an important class of diarrheagenic bacteria associated with severe diseases, such as hemorrhagic colitis and hemolytic uremic syndrome^12^. These *E. coli* strains are highly pathogenic to humans as they contain lambdoid prophages bearing genes coding for Shiga toxins (for a review, see^13^). Curiously, research on pathogenic EHEC bacteria allowed to identify sRNAs within bacteriophage-derived regions of the EHEC genome^14^. Although sRNAs in cryptic *E. coli* prophages (unable to produce viable progeny bacteriophages) were identified previously^15^,^16^], Tree *et al.* provided evidence for existence of such sRNAs within genomes of non-cryptic bacteriophages carrying Shiga toxin genes (*stx*). After prophage induction, the *stx* genes are efficiently expressed which results in production of relatively high amounts of Shiga toxins, the major agents responsible for high pathogenicity of EHEC strains^14^.

Shiga toxin-producing *E. coli* strains are of high interest as they cause local outbreaks, exemplified by quite a recent case which occurred in Germany in 2011 (for a review, see^17^). Moreover, treatment of patients infected with these bacteria is problematic, as many antibiotics stimulate induction of Shiga toxin-converting prophages, enhancing severity of the disease symptoms^18^. Therefore, identification of molecular mechanisms of regulation of induction and multiplication of these phages may facilitate development of novel therapeutic procedures. In the light of the recent discoveries described in the preceding paragraph, it is likely that lambdoid prophages of *E. coli* may encode various small regulatory RNAs, however, available data are limited, and most of them concerns a nonpathogenic λ phage which is a model member of lambdoid phage family (19,20], for review refer to^21^). Therefore, it seems that further complex research allowing for both identification of new sRNAs encoded in genomes of *stx*-bearing bacteriophages and assessment of their functions are still required.

Although extensive and diverse searches for sRNAs in variety forms of life have been carried out using different experimental methods, like microarray analysis, deep sequencing techniques or co-immunoprecipitation with Hfq, it is important to note that functions of only very limited number of the discovered sRNAs have been verified experimentally. On the other hand, some sRNAs with true regulatory functions might be still missed because they are only expressed under very specific conditions. For these reasons, we decided to search for, and determine functions of sRNAs encoded within the genome of the Φ24_B_ phage, one of the Shiga-toxin converting bacteriophages^22^,^23^,^24^, and produced after prophage induction during phage lytic development. We focused particularly on determination if microRNA-size molecules can be encoded by the bacteriophage, and if so, whether they can play specific regulatory role(s).

## Results

### Identification of a microRNA-size small RNA encoded by bacteriophage Φ24B

*E. coli* MG1655 bacteria lysogenic with Φ24_B_ phage were induced with mitomycin C (0.5 μg/ml), and small RNA molecules were extracted from samples withdrawn before (time 0) and 40 or 80 minutes after prophage induction. The determined by the Agilent 2100 Bioanalyzer concentrations of small RNA and microRNA (as a fraction of small RNA) corresponded to 5.6 (small RNA) and 2.0 (microRNA) pg/μl for samples collected at time zero, and 22.0 (small RNA) and 2.8 (microRNA) pg/μl, and 39.2 (small RNA) and 3.9 (microRNA) pg/μl for samples collected 40 and 80 minutes after prophage induction, respectively. The ratio of very small, miRNA-size molecules (10-40 nucleotides in length) to total isolated small RNAs, determined for analyzed samples, corresponded to 37%, 13% and 10% at times 0, 40 and 80 min, respectively. Both, the absolute microRNA content (in the range of pg/μl) and its percentage relative to small RNA (microRNA/small RNA ratio %) allowed for the identification and monitoring of microRNA fraction (range between 10–40 nt) among other small RNA species e.g. tRNA or rRNA (range between 40–150 nt). Obtained results were appropriate to proceed with preparation of cDNA libraries.

A cDNA library set of isolated sRNAs was prepared, and used for next generation sequencing (NGS). Data for samples withdrawn at times 0, 40 and 80 min after prophage induction consisted of 236459, 708614 and 700822 reads, respectively. The data derived from the material of the sample withdrawn 80 min after induction, allowed to identify 2223 reads of phage Φ24_B_ DNA sequence: TAA CGT TAA GTT GAC TCG GG, named by us 24B_1. It was the only phage-specific sequence found among all the reads. The remaining sequences were bacterial host-specific. Importantly, the generated data confirmed results obtained previously by other authors^7^ who first identified miRNA-size RNAs: EC-5p-36 and EC-3p-393 in *E. coli* (the reads corresponded to the previously reported sequences). To determine the genomic positions of the sequencing reads, the data were blasted to reference databases of Φ24_B_ phage (HM208303) and *E. coli* MG1655 (U00096) using Basic Local Alignment Search Tool (BLAST) and Clone Manager programs. Both reference databases were used for sRNAs mapping, however, since we focused on phage Φ24_B_, data obtained for *E. coli* were not further considered in this work.

**Figure 1:**
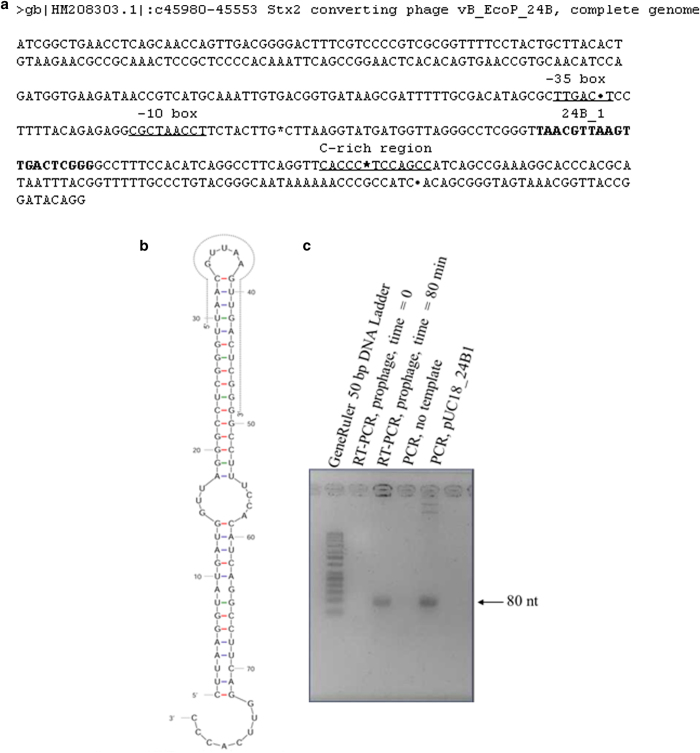
Localization and hairpin RNA structure of the identified 80-nt transcript and 24B_1 small RNA sequence. Panel **(a)** shows localization of the 24B_1 sequence (highlighted) within 428-nt long fragment (from residue 45553 to 45980) located between genes *lom* and *vb_24B_43* of Φ24_B_ phage genome. Underline is used to indicate localizations of boxes -10 and -35 of the predicted promoter *p*_24B-1_ as well as the C-rich site being a putative candidate for binding of the termination Rho protein. Bullets [•] are used to distinguish sequence removed by homologous recombination during MG1655 Φ 24_B_Δ24B_1 mutant construction. **(b)** Hairpin RNA structure predicted from the 80-nt long sequence indicated by asterisks [*] on panel (a). The 20-nt long sequence of 24B_1 small RNA is indicated within the predicted secondary structure by dotted line. (**c**) Detection of the 80-nt transcript, shown in panel (b) by RT PCR with templates isolated from lysogenic cells before (time = 0) or 80 min after (time = 80 min) prophage induction with 0.5 μg/ml mitomycin C). Control PCR reactions were performed with either no template (negative control) or with plasmid pUC18_24B1 (positive control).

The sequence of 20-nt long 24B_1 corresponds to residues 45711–45692 of the phage Φ24_B_ genome. It is located between genes *lom* and *vb_24B_43*. An *in silico* search for a possible promoter in this region revealed the presence of a candidate (Fig. 1a). The -10 and -35 boxes of this possible σ^70^- dependent promoter, named by us *p*_24B_1_,were CGCTAACCT and TTGACT, respectively, and the promoter score calculated using the BPROM software was 4.26 which indicates a high probability for active promoter^25^.

It was not possible to find a putative Rho-independent terminators within this 428 bp long sequence using ARNold, the online analysis tool which predicts the existence and location of Rho-independent transcription terminators employing RNAmotif and ERPIN complementary programs^26^,^27^,^28^,^29^. Searching within longer sequence fragment encompassing the whole *lom* gene was also unsuccessful. Data obtained by Peters *et al.* indicated that Rho-dependent terminators may be located at 3` ends of genes encoding small RNAs in *E. coli*^30^. Prediction of Rho-dependent terminators on the basis of DNA sequence is problematic because it is difficult to find common features for this kind of terminators. The only feature common to Rho-dependent terminators seems to be richness of C residues, as C-rich sites appear good candidates for binding of the Rho protein^31^,^32^,^33^. The sequence analysis allowed us to identify the C-rich region located downstream of the 24B_1 sequence which can suggest the presence of a Rho-dependent terminator (Fig. 1a).

Assuming functions of the predicted promoter and terminator, an 80-nt transcript should be produced. To test if this is true, we have designed specific primers for detection of such a transcript in RT-PCR. The lysogenic bacteria were treated with mitomycin C to induce the Φ24_B_ prophage, and RNA was isolated before and 80 min after induction. Following reverse transcription, the PCR with the specific primers was performed. No product could be observed at time 0, while the presence of the specific product, corresponding to the 80-nt transcript, was evident at time 80 min (Fig. 1c). The hairpin RNA structure of such transcript is presented in Fig. 1b, with indication of a 20-nt long 24B_1, detected experimentally as described above. We suggest that the 24B_1 RNA may be a result of the specific cleavage of the 80-nt transcript. This assumption can be supported by our observation that reads of sequences coming from non-specific degradation of other RNAs occurred in several forms of different lengths. However, in the case of the 24B_1 RNA, only one form of the 20-nt long sequence was identified in 2223 reads.

**Figure 2:**
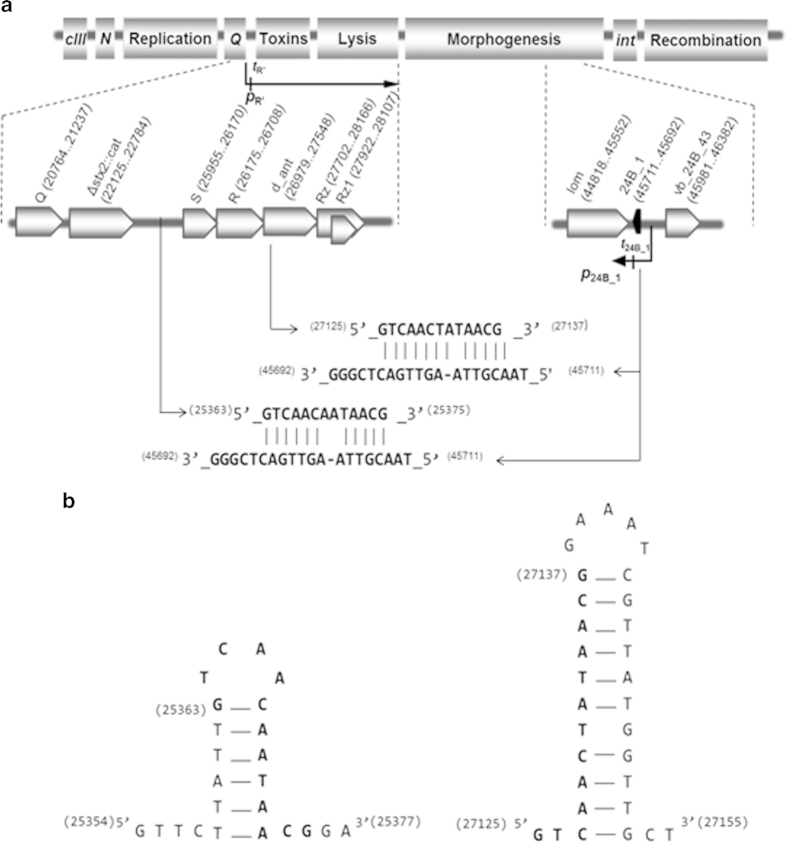
A schematic map of Φ24_B_ bacteriophage genome showing genomic locations of the 24B_1 sRNA, its potential binding sites (a) and their secondary structures (b). Regions of the genome which contain genes coding for proteins responsible for particular processes are indicated. Fragments of the Φ24_B_ genome responsible for late regulation and lysis as well as synthesis of the 24B_1 sRNA are enlarged and presented in the middle part of the panel (**a**). The most important promoters (*p*) and terminators (*t*) including those predicted for 24B_1 sRNA are indicated. Transcripts are presented as arrows with arrowheads indicating directionality of transcription. The complementarity regions between 24B_1 sRNA sequence and its two potential binding sites (first located upstream of the *S* gene and *vb_24B_23* open reading frame - not marked, and second located within the *d_ant* gene) are presented in the lower part of the panel (**a**). Sequences of both predicted binding sites are able to form secondary structures, which are presented in panel (**b**). Numbers in parentheses indicate positions within the Φ24_B_ genome.

Further *in silico* analyses indicated the presence of two potential binding sites for 24B_1 (Fig. 2a). One of them is located upstream of the *S* gene, and the second within the *d_ant* gene, whose sequence suggests that it can encode an anti-repressor protein. Sequences of both potential 24B_1 binding sites are able to form secondary structures, which are presented in Fig. 2b.

**Figure 3:**
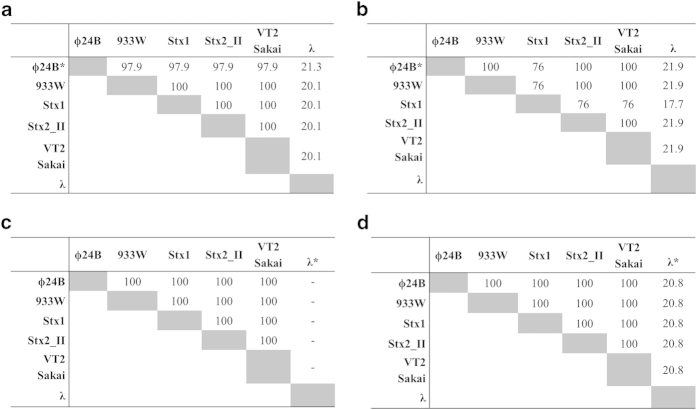
Scores of pairwise alignments of (a) 428-nt long sequences located downstream of the *lom* genes, (b) 700-nt long regions encompassing sequence of the first predicted binding site for 24B_1 sRNA located upstream of the *S* genes, (c) the *d_ant* gene sequences (in most cases called *ant*gene) located downstream of the *R* genes and (d) of the *S*gene sequences of all analyzed lambdoid phages: Φ24_B_ phage (HM208303), 933W phage (NC_000924), Stx1 converting phage (NC_004913), Stx2 converting phage II (NC_004914), VT2 Sakai phage (AP000422) and λ phage (NC_001416). Pairwise scores are simply the number of identities between the two sequences, divided by the length of the alignment, and represented as a percentage. The multiple sequence alignment was performed using the ClustalW algorithm. (**a**) * Note that the 20-nt long sequence of 24B_1 sRNA, the sequences -10 and -35 of the predicted promoter *p*_24B-1_ as well as the C-rich region of the putative *t*_24B_1_ Rho-dependent terminator sequence are absolutely conserved (100% identity) between all analyzed Shiga toxin-converting phages, however do not occur within analogous region of λ phage. (b) * Note that the sequence of the binding site is absolutely conserved (100% identity) between all analyzed Shiga toxin-converting phages, however does not occur within analogous region of λ phage. (**c**) *There is no *d_ant* (*ant*) gene located downstream of the *R* gene in case of λ phage. (**d**) *The sequences of λ *S* gene is completely different and longer (318 bp) in comparison with 216 bp long *S* gene sequences of analyzed Shiga toxin-converting phages. Nevertheless, two ATG codons were detected at 5’ end of each analyzed *S* gene.

Comparison of genomic sequences of phage Φ24_B_ and some other lambdoid phages strongly suggest that 24B_1 may be encoded by different Shiga toxin-converting bacteriophages, but not by bacteriophage λ (Fig. 3a). Similarly, both potential binding sites of 24B_1 share a high similarity between Shiga toxin-converting phages, while the region upstream of the *S* gene of phage λ is very different (Fig. 3b), and the *d_ant* gene is absent in this phage (Fig. 3c). In fact, the *S* genes of all analyzed Shiga toxin-converting phages are identical but with negligible similarity to the *S* gene of phage λ (Fig. 3d).

### Effects of the 24B_1 RNA on bacteriophage development

In order to test if the small, microRNA size, 24B_1 molecule can influence development of bacteriophage Φ24_B_, we have constructed a deletion mutant Φ24_B_Δ24B_1, lacking the 189–nt fragment encompassing the region shown in Fig. 1. In effect, the Φ24_B_Δ24B_1 mutant was unable to produce the studied sRNA. Measurement of efficiency of lysogenization indicated that the mutant phage forms prophages at significantly lower frequency relative to the wild-type counterpart (Table 1). Fractions of bacterial cells surviving the infection with Φ24_B_ and Φ24_B_Δ24B_1 were similar at m.o.i. = 1, however, significantly lower number of cells survived the infection of Φ24_B_Δ24B_1 relative to that of Φ24_B_ at m.o.i. = 5 and 10 (Table 2). These results indicate that lysogenization by phage Φ24_B_ is impaired in the absence of the 24B_1 RNA.

**Figure 4:**
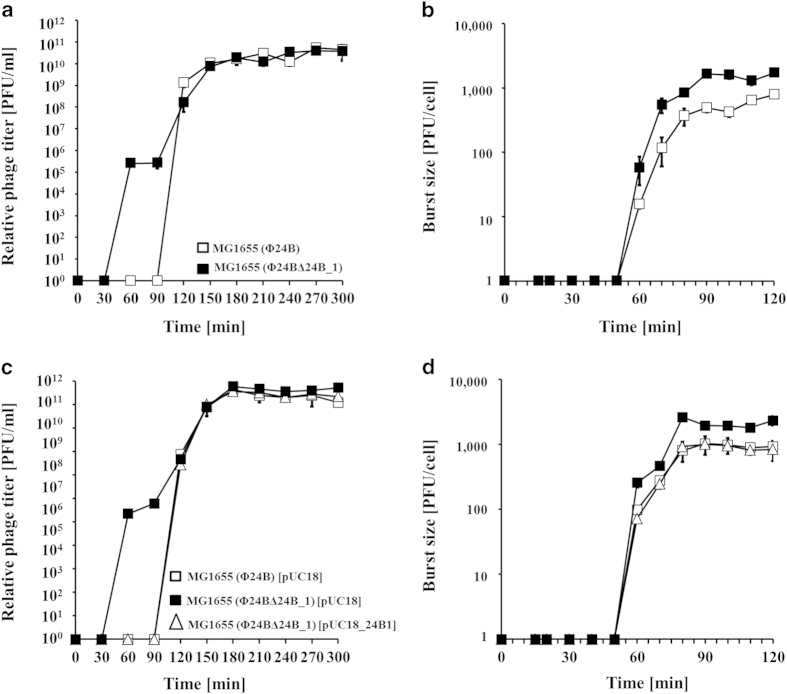
Development of Φ24_B_ and Φ24_B_∆24B_1 bacteriophages after prophage induction or phage infection at 37 ^o^C. *E. coli* MG1655 bacteria bearing no plasmid (□, ■ in panels **a** and **b**), plasmid pUC18 (□, ■ in panels **c** and **d**) or plasmid pUC18_24B1 (∆ in panels **c** and **d**) were hosts for phage development. The hosts were either lysogenic for Φ24_B_ (□ in panels **a** and **c**) or Φ24_B_∆24_B__1 (■, ∆ in panels **a** and **c**), or infected with Φ24_B_ (□ in panels **b** and **d**) or Φ24_B_∆24_B__1 (■, ∆ in panels **b** and **d**). Phage lytic development was initiated by addition of mitomycin C to final concentration of 1 μg/ml (panels **a** and **c**) or phage infection (panels **b** and **d**) at time 0. The presented results are mean values from three independent experiments with error bars indicating SD (note that in the most cases, the bars are smaller than sizes of symbols). Results are shown as PFU (plaque forming units) per one ml of bacterial culture **(a, c)** or per cell **(b, d)**.

No significant difference in spontaneous prophage induction was noted between Φ24_B_ and Φ24_B_Δ24B_1 lysogens, which was at the range of 10^−4^ per cell. However, addition of mitomycin C (up to 1 μg/ml) to provoke the SOS response and subsequent prophage induction resulted in significantly more rapid switch from lysogeny to lytic phage development by Φ24_B_Δ24B_1 than Φ24_B_, though the final efficiency of progeny phage production, as well as host cell lysis (as measured by a decrease in the density of bacterial culture) were similar in both cases (Fig. 4a).

**Figure 5:**
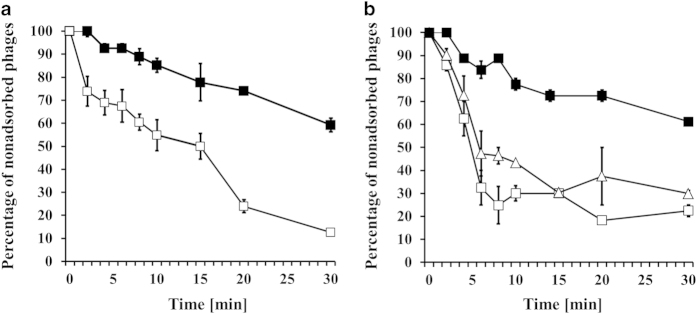
Adsorption of Φ24_B_ and Φ24_B_∆24B_1 bacteriophages on *E. coli*cells at 37 ^o^C. *E. coli* MG1655 bacteria bearing no plasmid (□, ■ in panel **a**), plasmid pUC18 (□, ■ in panel **b**) or plasmid pUC18_24B1 (∆ in panel **b**) were used. Phage Φ24_B_ (□ in panel **a** and **b**) or Φ24_B_∆24B_1 (■, ∆ in panels **a** and **b**) were added to bacterial cell suspension, and percent of nonadsorbed virions was estimated at indicated times. The presented results are mean values from three independent experiments with SD indicated by error bars.

Phage lytic development, following infection of non-lysogenic *E. coli,* was more efficient in Φ24_B_Δ24B_1-infected bacteria relative to Φ24_B_, though kinetics of appearance of progeny viruses was similar in both experiments (Fig. 4b). Interestingly, we found that adsorption of Φ24_B_Δ24B_1 virions on the host cells is significantly impaired relative to the wild-type phage (Fig. 5).

To test if all these physiological effects observed in the Φ24_B_Δ24B_1 mutant arise from the lack of the 24B_1 RNA, rather than from any polar effects of the deletion made in the phage genome, we have constructed a plasmid bearing the bacteriophage Φ24_B_ DNA fragment encompassing the region which was deleted in the mutant phage. Phenotypes of Φ24_B_ and Φ24_B_Δ24B_1 bacteriophages in bacterial hosts bearing the vector (pUC18 plasmid) were indistinguishable from those found in plasmid-less bacteria (Tables 1,2, and Figs. 4,5). However, in cells bearing a plasmid with the cloned phage DNA fragment, all tested phenotypes of Φ24_B_Δ24B_1 were very similar (without statistically significant differences) to those of the wild-type phage (Tables 1,2, and Figs. 4,5). These results indicate that *in trans* provision of the DNA fragment which is lacking in the Φ24_B_Δ24B_1 mutant phage can fully complement the physiological effects of the deletion. Therefore, the changes in the mutant phage development cannot be due to any polar effects of the deletion.

### Bacteriophage gene expression patterns in the presence and absence of the 24B-1 RNA

**Figure 6:**
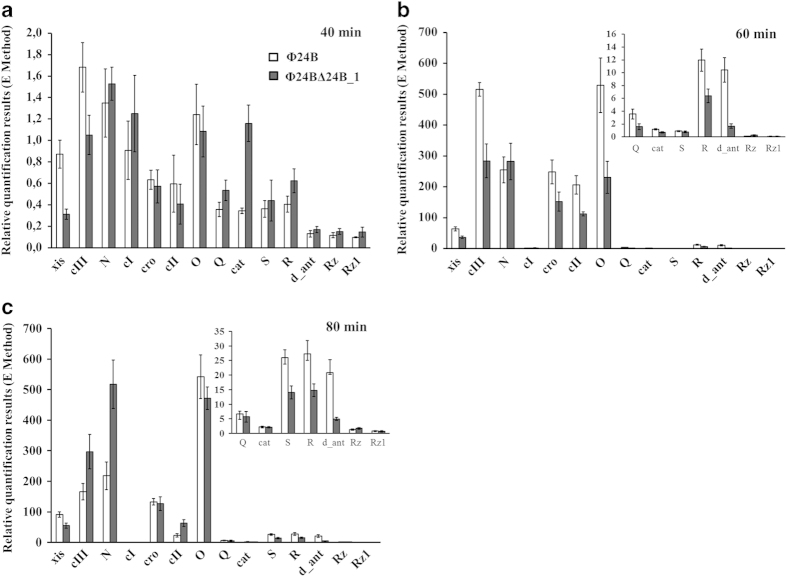
Levels of transcripts of indicated genes of Φ24_B_(□) and Φ24_B_∆24B_1 (■) bacteriophages assessed by quantitative reverse RT-PCR analysis, after prophage induction with 1 μg/ml mitomycin C in *E. coli* MG1655 host at 37 ^o^C. Levels of transcripts corresponding to particular genes were determined at following times after induction: 40 **(a)**, 60 **(b)**, or 80 **(c)** minutes. Results are presented as mean values from three independent experiments with error bars indicating SD.

To test if 24B_1 RNA can affect expression of phage Φ24_B_ genes, levels of particular mRNAs were monitored after prophage induction or bacteriophage infection by using the quantitative reverse transcription real time PCR (qRT PCR). Following prophage induction, only minor differences could be observed in efficiency of expression of most tested phage genes between Φ24_B_ and Φ24_B_Δ24B_1 (Fig. 6). At 40 min after induction, tested genes were expressed at low levels in both hosts. Although some differences were found between tested strains in the levels of xis, cIII, cat and R transcripts, one should note that these differences were small in real values (note different values at Y scales in panels a, b, and c of Fig. 6). Nevertheless, levels of transcripts derived from *c*III, *cro, c*II and *O* genes were higher in cells bearing Φ24_B_ relative to Φ24_B_Δ24B_1 at 60 min after addition of mitomycin C, while *c*III and *N* gene were expressed more efficiently in the mutant phage at the later time (Fig. 6).

**Figure 7:**
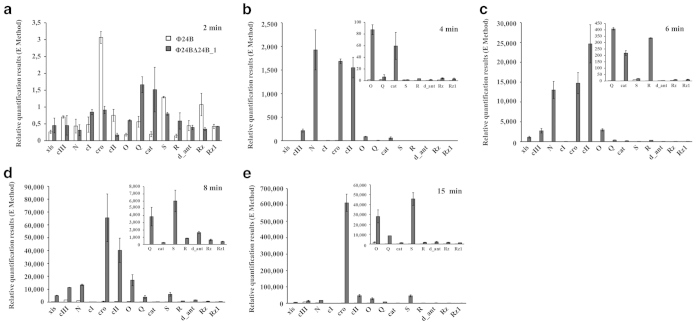
Expression patterns of indicated genes of Φ24_B_ (□) and Φ24_B_∆24B_1 (■) bacteriophages infecting *E. coli* MG1655 host at 37 ^o^C, assessed by quantitative reverse RT-PCR analysis. Levels of transcripts corresponding to particular genes were determined at following times after infection: 2 **(a)**, 4 **(b)**, 6 **(c)**, 8 **(d)** or 15 **(e)** minutes. The presented results are mean values from three independent experiments with error bars indicating SD.

Contrary to lysogenic cells, drastic differences in levels of phage mRNAs were observed in bacteria infected with Φ24_B_ and Φ24_B_Δ24B_1 shortly after infection (Fig. 7). Although 2 min after infection expression of all phage genes was negligible, at times between 4^th^ and 15^th^ min, expression of all tested genes, both early (from *p*_L_ and *p*_R_ operons, and the *c*I gene) and late (from the *p*_R’_ operon), was drastically increased in Φ24_B_Δ24B_1- infected cells relative to those infected with Φ24_B_ (Fig. 7).

## Discussion

Although small RNAs are considered to be major regulatory elements in eukaryotic cells, our knowledge on such regulations in prokaryotic systems is less advanced. This concerns especially microRNA-size molecules. Small RNA fragments, with length between 15 and 28 nt, were identified in *E. coli*^7^, however, no functional studies with these RNAs were reported. The first candidate for microRNA of bacterial origin was discovered recently^10^, but this 23-nt small RNA, produced by *Mycobacterium marinum,* affects expression of the eukaryotic host, rather than bacterial, genes. Therefore, we aimed to test if microRNA-size molecules can modulate expression of prokaryotic genes and be of physiological significance. As a model, we used bacteriophage Φ24_B_, a lambdoid phage carrying Shiga toxin genes. In fact, evidence for existence of small RNAs encoded within genomes of other Shiga toxin-converting phages has been demonstrated^14^, however, this did not concern microRNA-size molecules.

Our NGS analysis of small RNA molecules indicated the existence of a 20-nt long RNA encoded by bacteriophage Φ24_B_. This RNA likely derives from a longer, about 80-nt long transcript which appears to initiate at the promoter located between genes *lom* and *vb_24B_43* of Φ24_B_ phage genome (Fig. 1). We named this microRNA-size molecule 24B_1. Since it appeared quite abundant in the NGS analysis, we assume that 24B_1 is a product of specific cleavage of the longer transcript. This would resemble formation of micro-RNAs in eukaryotic cells, and suggest that 24B_1 might be formally considered as this type of sRNA.

The crucial question was whether 24B_1 has any physiological role. Therefore, we tested various aspects of development of bacteriophage Φ24_B_ bearing deletion of the region encoding the microRNA-size precursor. Interestingly, we found that lysogenization of the host cells by the mutant phage is less efficient than by wild-type phage. Induction of the mutant prophage was quicker, and lytic development more efficient than in wild-type phage. Interestingly, the phage lacking 24B_1 adsorbed less efficiently on the host cell. All these differences were not dramatic, but significant.

In order to learn on the mechanism(s) of 24B_1-mediated regulation, we investigated expression of crucial phage genes in the mutant and wild-type phages either after prophage induction or after infection of the host. Our *in silico* analysis revealed that 24B_1 may potentially bind to two sites, one located upstream of the *S* gene, and the second within the *d_ant* gene (Fig. 2a). The latter gene encodes a putative anti-repressor protein, as its sequence is identical to anti-repressor genes (called *ant*) in some other lambdoid phages (Fig 3c). Our RT-qPCR analysis indicated that there are some differences in expression of *N*, *c*III, *cro, c*II and *O* genes between the mutant and wild-type phages. However, dramatic differences were observed between both these phages after infection of the host cells. Expression of all tested phage genes was drastically increased in the cells infected with the phage lacking 24B_1, relative to the wild-type phage. These differences in gene expression efficiency can explain most, if not all, phenotypes observed for the mutant phage. The most straight forward explanation would be the action of the 24B_1 microRNA-size molecule as a negative regulator of the *d_ant* gene expression. In the mutant phage, expression of the gene coding for the antirepressor would be enhanced, resulting in more efficient inhibition of the cI repressor. Under such conditions, expression of most phage genes would be enhanced, as this repressor is a negative regulator of major phage promoters. Physiological effects of such regulation, observed in the mutant phage, would be impaired lysogenization of the host cells (due to less efficient repression of the “lytic” promoters), quicker prophage induction, and more efficient lytic development. Less effective phage adsorption on the host cells might result from unbalanced expression of structural phage proteins, and formation of an increased number of partially defective virions.

In conclusion, we have demonstrated the existence of a microRNA-size molecule derived from phage Φ24B in *E. coli* cells. This small RNA, named 24B_1, has a physiological role, as the mutant phage lacking the region encoding its precursor revealed dramatic changes in expression of all tested phage genes, and significant differences in various developmental processes. To our knowledge, this is the first demonstration of physiological significance of a microRNA-sized molecule in bacterial cells.

## Materials and methods

### Bacteria, bacteriophages and plasmids

*E. coli* MG1655 strain^34^ was the host of choice for lysogenization, bacteriophage infection and prophage induction experiments. Bacteria were routinely cultured in the LB medium. Phages Φ24_B_ (∆*stx2*::*cat*)^22^ and Φ24_B_Δ24B_1 (this work) were employed. The deletion mutant MG1655Φ24_B_Δ24B_1 (lacking the identified sRNA species, named 24B_1) was constructed using the Quick and Easy *E. coli* Gene Deletion Kit (from Gene Bridges). The deletion of 189–nt long region of Φ24_B_ prophage genome (encompassing the identified 24B_1 sequence, as well as the 80-nt long sequence of the predicted secondary structure) was performed according to the manufacturer’s protocol using following primers: pFΔ24B_1: 5` CAT TGG CCT GAA ATT CTG ACC TGT ATC CGG TAA CCG TTT ACT ACC CGC TGT AAT TAA CCC TCA CTA AAG GGC G 3`, and pR Δ24B_1: 5` TCA TGC AAA TTG TGA CGG TGA TAA GCG ATT TTT GCG ACA TAG CGC TTG ACT AAT ACG ACT CAC TAT AGG GCT C 3`. Using this kit, in the first step, we replaced the targeted sequence with the FRT-flanked kanamycin resistance cassette and subsequently removed the selection marker by a FLP-recombinase step, leaving only 87 nucleotides of the cassette in the place of the 189–nt long original sequence, what was confirmed by DNA sequencing. The deleted region was distinguished from the sequence presented in Fig. 1a by bullets [•].

Bacteriophage suspensions were routinely stored in the TM buffer (10 mM Tris-HCl, 10 mM MgSO_4_, pH 7.2) at 4 ^o^C.

For construction of plasmid pUC18_24B1, the region from phage Φ24_B_ DNA encompassing the 80-nt long sequence of the predicted secondary structure of 24B_1 was amplified by PCR with primers F24B1_EcoRI (5′ TCT GAA TTC ACG CTG CAT ATA CCG GAG AA 3′) and R24B1_HindIII (5′ GTG GAA GCT TTG GAT GTG GCT TAC GAA GGT 3′), and the phage genome as a template (phage Φ24_B_ DNA was isolated using MasterPure™ Complete DNA and RNA Purification Kit; Epicentre). Following digestion with EcoRI and HindIII, the amplified region was ligated with the EcoRI-HindIII fragment of plasmid pUC18 (Thermo Fisher Scientific Inc., Waltham, MA, USA) bearing an ampicillin resistance gene. The construction of pUC18_24B1 was confirmed by DNA sequencing.

### Small RNAs extraction

Three sets of small RNA molecules of *E. coli* MG1655 strain lysogenic for Φ24_B_ phage were extracted from samples withdrawn before (time 0) and at indicated times after prophage induction. To induce lytic development, mitomycin C (0.5 μg/ml) was added at A_600 _= 0.1. sRNAs were extracted from equal amount of cells (5 × 10^6^) using PureLink miRNA Isolation Kit (Life Technologies), designed to isolate high quality small RNAs. The isolation was performed according to the manufacturer’s protocol. The concentration of the purified small RNAs was evaluated using Qubit RNA Assay Kit as well as Qubit miRNA Assay Kit (both provided by Life Technologies). The quality and quantity of the isolated small RNAs, as well as the composition of the small RNA fractions were monitored using Agilent 2100 Bioanalyzer.

### cDNA libraries’ preparation

Preparation of cDNA libraries from the isolated microRNA fractions was performed using the NEBNext Small RNA Library Prep Set for Illumina (New England Biolabs), which allows to convert small RNA transcripts into barcoded cDNA libraries, suitable for next-generation sequencing on the Illumina platform. The isolation of the microRNA fraction form the gel was performed according to protocols of Genomed S.A.

### DNA sequencing

Next Generation Sequencing (NGS) of prepared cDNA libraries was performed using MiSeq (Illumina) Genome Sequencer according to contractor’s protocols. Three sets of sequence data, derived from materials withdrawn at times indicated above (0, 40 and 80 min) were processed by Genomed S.A. using Illumina’s software.

### *In silico* analyses

Prediction of putative promoters in the genomic sequence of the phage Φ24_B_ was performed using BPROM – the bacterial σ^70^ promoter recognition program available at: http://linux1.softberry.com/berry.phtml?topic=bprom&group=programs&subgroup=gfindb

Promoters were searched within ~428 bp long sequence fragment (from base 45553 to 45980) located between genes *lom* and *vb_24B_43* and encompassing the 20 nucleotide-long sequence of 24B_1 sRNA. BPROM has accuracy of *E. coli* promoter recognition about 80%, and considers promoters with score above 0.20^25^.

A search for putative Rho-independent terminators was performed using ARNold, the online analysis tool which predicts the existence and location of such terminators employing RNAmotif, and ERPIN complementary programs^26^,^27^,^28^,^29^. The ARNold program is available at: http://rna.igmors.u-psud.fr/toolbox/arnold/.

The hairpin RNA structure was predicted using Mfold software^35^. The pairwise as well as multiple sequence alignments were performed using the ClustalW algorithm available at the website: http://www.genome.jp/tools/clustalw/.

### Detection of the 80-nt transcript

Isolation of microRNA was performed by using the PureLink miRNA Isolation Kit (Life Technologies), according to the manufacturer’s instruction. DNA was removed and reverse transcription reaction was performed as described below (subsection “Preparation of RNA and cDNA from phage-infected bacteria and from lysogenic bacteria after prophage induction”). For PCR amplification with StartWarm 2 x PCR Master Mix (A&A Biotechnology), primers pF_80nt_24B1 and pR_ 80nt_24B1 (Table 3) were employed. Amplification products were separated and visualized by agarose gel electrophoresis.

### Prophage induction experiment

Bacteria lysogenic for tested phages were cultured in Luria–Bertani (LB) medium at 37 °C to A_600_ of 0.2. Induction of prophages was provoked in lysogenic bacteria by addition of mitomycin C to a final concentration of 1 μg ml^−1^. Following induction, at indicated times, 0.5-ml samples were withdrawn. Then, 30 μl of chloroform was added to each sample, the mixture was vortexed and centrifuged for 5 min in a microcentrifuge. The phage lysate was titrated on *E. coli* MG1655 host. Serial dilutions were prepared in TM buffer (10 mM Tris–HCl, 10 mM MgSO_4_; pH 7.2). Phage titer (number of phages per ml) was determined by spotting 2.5 μl of each dilution of the phage lysate on a freshly prepared LB agar (1.5%) with 2.5 μg/ml chloramphenicol, to obtain visible plaques formed on bacterial lawn (according to a procedure described by^36^), with a poured mixture of 1-ml indicator *E. coli* MG1655 strain culture and 2 ml of 0.7% nutrient agar (prewarmed to 45 °C), supplemented with MgSO_4_ and CaCl_2_ (to a final concentration of 10 mM each). Plates were incubated at 37 °C overnight. The relative phage titer (PFU/ml) was normalized to results of control experiments (representing ratios of phage titers in induced and non-induced cultures). Each experiment was repeated three times.

### One-step-growth experiment

Intracellular phage lytic development was studied in one-step-growth experiments. Bacteria were grown in LB medium supplemented with MgSO_4_ and CaCl_2_ (to a final concentration of 10 mM each) at 37 °C to A_600 _= 0.2. Samples of 10 ml were withdrawn and centrifuged (3,000×*g*, 10 min). Each pellet was suspended in 1 ml (1/10 of initial volume) of 3 mM NaN_3_ in LB. Following 5-min incubation at 37 °C, the phage was added to multiplicity of infection (m.o.i.) of 0.05. Phage adsorption was carried out at 37 °C for 10 min. The mixture was diluted ten-fold in warm (37 °C) 3 mM NaN_3_ in LB and centrifuged (3,000×*g*, 10 min). Bacterial pellet was suspended in 1 ml of LB with 3 mM NaN_3_ and centrifuged again (3,000×*g*, 10 min). This procedure was repeated three times. The suspension was then diluted 1,000-fold with LB, prewarmed to 37 °C (time 0), and aerated in a water bath shaker at this temperature. The number of infected bacteria was determined as follows. Culture samples were withdrawn at times between 0 and 15 min after infection. 0.01 ml of each serial dilution of such samples was mixed with 1 ml of an overnight culture of *E. coli* strain MG1655. Then, 2 ml of the top agar was added, mixed and poured onto an LB plate with 2.5 μg/ml chloramphenicol (according to a procedure described by^36^). After overnight incubation at 37 ^o^C, the number of plaques was determined. Infected cells were named “infection centers” [IC] (they were sources of new phages, which were released from host cells after one lytic cycle, and following infection of neighboring cells could form plaques). Samples withdrawn at later times were shaken vigorously for 1 min with 30 μl of chloroform, cleared by centrifugation and titrated to determine the number of PFU (number of phages able to form plaques) per ml. Plates were incubated at 37 °C overnight. Burst size was calculated as a ratio of phage titer to the titer of infection centers. Each experiment was repeated three times.

### Measurment of the efficiency of phage adsorption

*E. coli* MG1655 host cells were grown in LB medium at 37 °C to A_600_ = 0.4. Samples of 6 ml were centrifuged and pellets were washed with 1 ml of 0.85% NaCl. After centrifugation, each pellet was suspended in 1.5 ml LB medium supplemented with MgSO_4_ and CaCl_2_ (to a final concentration of 10 mM each). Tested bacteriophages were added to m.o.i. (multiplicity of infection) of 0.1 and the mixtures were incubated at 37 ^o^C. During the incubation, 0.1-ml samples were withdrawn at indicated times, centrifuged (6,000 x g for 1 min at room temperature) and the supernatant was titrated. Plates were incubated at 37 °C overnight. Each experiment was repeated three times. A sample withdrawn immediately after addition of bacteriophages to the cell suspension (time zero) was considered as 100% non-adsorbed phages. Other values were calculated relative to this value.

### Efficiency of lysogenization

The procedure described previously was used, with slight modifications^37^. Briefly, host bacteria were cultured to A_600 _= 0.5 in LB medium supplemented with MgSO_4_ and CaCl_2_ (to final concentrations of 10 mM each) at 37 °C with shaking. Cultures were washed with TCM buffer (10 mM Tris-HCl pH 7.2, 10 mM MgSO_4_, 10 mM CaCl_2_) twice, and then pellets were suspended in the same buffer. Aliquots of these cultures were mixed with phage suspensions at multiplicity of infection (m.o.i.) = 1, 5, or 10 in a final volume of 200 μl. Mixtures of bacteria and phages were incubated in TMC buffer for 30 min at 37 ^o^C, then one half of each mixture was spread on LB agar plates (control) and the second half on LB agar plates containing 20 μg/ml chloramphenicol (presumptive lysogens). Efficiency of lysogenization was calculated as a percent of lysogens among all bacterial cells (determined on the basis of number of colonies appearing on LB agar plates with no antibiotic). Lysogens were verified by testing resistance to superinfection by the same phage and sensitivity to UV irradiation, as described previously^38^. Each experiment was repeated three times.

### Survival of cells after bacteriophage infection

To estimate the percentage of surviving cells after bacteriophage infection, host bacteria were grown in LB medium at 37 °C to A_600 _= 0.3. 4 ml volume was centrifuged (2,000 x g for 10 min at 4 ^o^C) and the pellet was washed with 1-ml of 0.85% NaCl. After centrifugation, each pellet was suspended in 1.2 ml of LB medium supplemented with MgSO_4_ and CaCl_2_ (to a final concentration of 10 mM each) and cultures were incubated for 30 min at 37 ^o^C. Tested bacteriophages were added to m.o.i of 1, 5 or 10. The mixture was incubated at 37 ^o^C for next half an hour. Following incubation, serial dilutions in TM buffer (10 mM Tris–HCl, 10 mM MgSO_4_; pH 7.2) were prepared and 30 μl of each dilution was spread on LB agar plates. Plates were incubated at 37 °C overnight. Percentage of surviving bacteria was calculated relative to parallel sample with addition of TM buffer instead of bacteriophage lysate. Each experiment was repeated three times.

## Preparation of RNA and cDNA from phage-infected bacteria and from lysogenic bacteria after prophage induction

Bacterial culture was grown to A_600_ of 0.3 at 37 ^o^C. 120 ml volume was centrifuged and then the pallet was washed with 30 ml of 0.85% NaCl. After centrifugation, samples were suspended in 36 ml of LB medium enriched by MgSO_4_ and CaCl_2_ (to a final concentration of 10 mM each). The mixture was incubated for 30 min at 37 ^o^C and chilled on ice. Tested bacteriophage lysate was added to m.o.i. of 1.5. Following incubation on ice, infected bacterial cells were aerated in a water bath shaker at 37 ^o^C. At indicated times, 1 × 10^9^ samples were treated with NaN_3_ (Sigma-Aldrich) to a final concentration of 10 mM and harvested.

The induction of temperate bacteriophages from *E. coli* strain MG1655 was performed with mitomycin C, added to a final concentration 1 μg/ml. Following induction, the growth of bacteria was inhibited at indicated times by the addition of NaN_3_ (Sigma-Aldrich) to a final concentration of 10 mM.

Total RNA was isolated from 1 × 10^9^ bacterial cells using the High Pure RNA Isolation Kit (Roche Applied Science). Bacterial genomic DNA carryover was removed by incubation with TURBO™ DNase from TURBO DNA-free™ Kit (Life Technologies) for 60 min at 37 ^o^C according to the manufacturer’s guidelines. Then, RNA was quantified using Qubit® RNA BR Assay Kit (Invitrogen) and Qubit® 2.0 Fluorometer (Life Technologies), which provides an accurate and selective method for the quantitation of high-abundance RNA samples. The band patterns of total RNA were also visualized by electrophoresis. The absence of DNA from RNA samples was controlled by PCR amplification and by real-time PCR amplification (all analyzed genes were tested). RNA preparations were stored at −80 ^o^C for further use. cDNA from the total RNA samples (1.25 μg) was obtained with Transcriptor Reverse Transcriptase and random hexamer primers (Roche Applied Science) following the instructions supplied from the provider. cDNA reaction mixtures were diluted 10-fold for use in real-time PCR.

### Real-time PCR assay

For transcriptional analysis of tested genes by quantitative real-time reverse transcription-PCR (qRT-PCR) the qRT-PCR was performed using the LightCycler® 480 Real-Time PCR System (Roche Applied Science) with cDNA samples from lysogenic bacteria (as described previously^39^). Transcription rates of tested genes were compared in parallel to the *icdA* housekeeping gene (according to a previous report^40^). Specific oligonucleotide primers were developed by Primer3web version 4.0.0 and produced by Sigma-Aldrich or GENOMED. The transcriptional analysis of Φ24_B_ and Φ24_B_Δ24B_1 genes were performed with primers presented in Table 3. Real-time PCR amplifications were performed for 55 cycles in 20-μl reaction volumes by using LightCycler® 480 SYBR Green I Master (Roche Applied Science). Reactions were performed in Roche 96-well plates containing 10 μl 2 x SYBR Green I Master Mix, 6.25 ng/μl cDNA and 200 nM of each gene-specific primers (Table 3). Relative quantification assays were performed with cDNA in an *icdA* and phage genes multiplex assay. For the tested genes the cycling conditions were: 95 ^**o**^C for 5 min; 55 cycles of 95 ^**o**^C for 10 s; 60 ^**o**^C for 15 s and 72 ^**o**^C for 15 s. No template control was included with each run. Each reaction was repeated three times. The specificity of amplified products was examined by melting curve analysis immediately after the final PCR cycle and confirmed by gel electrophoresis.

### Real-time data analysis

The relative changes in gene expression revealed by quantitative Real-Time PCR experiments were analyzed using the calibrator normalized relative quantification method with efficiency correction (as described previously by^39^). This method is called E-Method and provides an efficiency corrected calculation mode by using the determined PCR efficiency of target (E_t_) as well as the efficiency of reference (E_r_). Relative fold change ratio was calculated by using the formula described in the application manual of Roche LightCycler Real-Time PCR Systems (see ref.[40]). The Roche formula for the normalized relative ratio (NRR) was described as follows: NRR = E_t_^CT(target) calibrator – CT(target) sample^ /E_r_^CT(reference) calibrator – CT(reference) sample^. The sample at the time point “zero“ was a calibrator. The raw run data for tested phage genes were transferred from the LightCycler 480 to the LinRegPCR 12.5 software using the “LC480 Conversion: conversion of raw LC480 data” software (available at http://www.hartfaalcentrum.nl/index.php?main=files&sub=0.php?main=files&sub=0). PCR efficiency was determined for each gene by employing the LinRegPCR software which was successfully used to calculate PCR efficiency previously^41^,^42^,^43^,^44^,^45^,^46^,^47^.

## Author Contributions

Conceived and designed the experiments: B.N.F., S.B., A.W. and G.W. Performed the experiments: B.N.F., S.B., K.L., A.D. and G.T. Analyzed the data: B.N.F., S.B., A.F., A.W. and G.W. Contributed reagents/materials/analysis tools: B.N.F., S.B., A.W. and G.W. Contributed to the writing of the manuscript: B.N.F., S.B., A.W., G.W. B.N.F. and S.B. contributed equally to this work.

## Additional Information

**How to cite this article**: Nejman-Faleńczyk, B. *et al*. A small, microRNA-size, ribonucleic acid regulating gene expression and development of Shiga toxin-converting bacteriophage Ф24_B_. *Sci. Rep.* **5**, 10080; doi: 10.1038/srep10080 (2015).

## Figures and Tables

**Table 1:** Efficiency of lysogenization of *E.*

**Strain**	**Efficiency of lysogenization (% of lysogenes among survivors)**					
	**Phage Φ24**_**B**_			**Phage Φ24**_**B**_ **∆24** **B** **_1**		
	**m.o.i.=1**	**m.o.i.=5**	**m.o.i.=10**	**m.o.i.=1**	**m.o.i.=5**	**m.o.i.=10**
MG1655	18.5 ± 0.8	65.9 ± 6.1	71.6 ± 4.4	9.7 ± 1.8	45.7 ± 8.2	54.8 ± 5.0
MG1655 [pUC18]	16.6 ± 1.2	63.6 ± 4.2	84.6 ± 8.0	10.1 ± 2.0	41.5 ± 3.6	50 ± 2.5
MG1655 [pUC18_24B1]	nt	nt	nt	24 ± 3.5	57.1 ± 8.5	76.8 ± 8.4

*coli* wild-type MG1655, MG1655 [pUC18] and MG1655 [pUC18_24B1] strains with lambdoid bacteriophages: Φ24_B_ and Φ24_B_∆24B_1. Results are presented as mean values from three independent experiments ± SD. Statistical analysis (*t* test) was performed for results from each m.o.i. (multiplicity of infection), and indicated significant differences (*P* < 0.05) between efficiency of lysogenization of host cell by both tested bacteriophages in all cases except the variants of complementation (MG1655 or MG1655 [pUC18] hosts infected with Φ24_B_ versus MG1655 [pUC18_24B1] infected with Φ24_B_∆24_B__1) where no significant differences (*P* > 0.05) were found. nt – not tested.

**Table 2:** Survival (%) of the wild-type host strain *E.*

**Strain**	**Survival of cells in infected culture (% of survivors)**					
	**Phage Φ24**_**B**_			**Phage Φ24**_**B**_ **∆24** **B** **_1**		
	**m.o.i.=1**	**m.o.i.=5**	**m.o.i.=10**	**m.o.i.=1**	**m.o.i.=5**	**m.o.i.=10**
MG1655	71 ± 9	49 ± 9	65 ± 8	61 ± 12	22 ± 15	31 ± 0
MG1655 [pUC18]	85 ± 6.3	52 ± 6.3	75 ± 8.3	77 ± 2.1	21 ± 4.0	17 ± 3.8
MG1655 [pUC18_24B1]	nt	nt	nt	81 ± 2.1	50 ± 8.3	60 ± 2.1

*coli* MG1655, MG1655 [pUC18] and MG1655 [pUC18_24B1] after infection with Φ24_B_ and Φ24_B_∆24B_1 bacteriophages. Mean values from three independent experiments ± SD are shown. Statistical analysis (*t* test) was performed for results from each m.o.i. and indicated significant differences (*P* < 0.05) between fractions of bacterial cells surviving the infection with Φ24_B_ and Φ24_B_∆24_B__1 at m.o.i = 5 and 10 in most cases except the variants of complementation (MG1655 or MG1655 [pUC18] hosts infected with Φ24_B_ versus MG1655 [pUC18_24B1] infected with Φ24_B_∆24_B__1) where no significant differences (*P* > 0.05) were found. Differences in the mean fold change values for m.o.i = 1 between tested bacteriopohages did not reach statistical significance in the *t* test with *P* > 0.05. nt – not tested.

**Table 3:** Primers used in PCR and real time PCR assays.

**Primer name**	**Sequence (5’→ 3’)**
pF_Φ24B_xis pR_ Φ24B_xis	TATCGCGCCGGATGAGTAAG CGCACAGCTTTGTATAATTTGCG
pF_Φ24B_cIII pR_ Φ24B_cIII	ATTCTTTGGGACTCCTGGCTG GTAAATTACGTGACGGATGGAAAC
pF_Φ24B_N pR_ Φ24B_N	AGGCGTTTCGTGAGTACCTT TTACACCGCCCTACTCTAAGC
pF_Φ24B_cI pR_ Φ24B_cI	TGCTGTCTCCTTTCACACGA GCGATGGGTGGCTCAAAATT
pF_Φ24B_cro pR_ Φ24B_cro	CGAAGGCTTGTGGAGTTAGC GTCTTAGGGAGGAAGCCGTT
pF_Φ24B_cII pR_ Φ24B_cII	TGATCGCGCAGAAACTGATTTAC GACAGCCAATCATCTTTGCCA
pF_Φ24B_O pR_ Φ24B_O	AAGCGAGTTTGCCACGAT GAACCCGAACTGCTTACCG
pF_Φ24B_Q pR_ Φ24B_Q	GGGAGTGAGGCTTGAGATG TACAGAGGTTCTCCCTCCCG
pF_Φ24B_cat pR_ Φ24B_cat	TCACCAGCTCACCGTCTTTC TTCTTGCCCGCCTGATGAAT
pF_Φ24B_S pR_ Φ24B_S	CTGGGGAGTCTGCTGTTTGG GCCTTACGCCGGTCTTCTTT
pF_Φ24B_R pR_ Φ24B_R	GGGTGGATGGTAAGCCTGT TAACCCGGTCGCATTTTTC
pF_Φ24B_d_ant pR_ Φ24B_d_ant	TGTTTGCTACCGGGCTGAAT CCCTTTGCCTGTATCAGCCA
pF_Φ24B_Rz pR_ Φ24B_Rz	AACCGTGTTCTGTGTGTGGT GGTTTGTTGCCAGCCACAG
pF_Φ24B_Rz1 pR_ Φ24B_Rz1	CGGATCAACGCCACCTGC GTTGCATTATCCACGCCGG
pF_Φ24B_icdA pR_ Φ24B_icdA	CGAAGCGGCTGACCTTAATTG GTTACGGTTTTCGCGTTGAT
pF_80nt_24B1 pR_ 80nt_24B1	CTTAAGGTATGATGGTTAGGGCCTCG GGGTGAACCTGAAGGCCTGATG
